# Book Review: Anti-VEGF Use in Ophthalmology

**DOI:** 10.1186/s40942-017-0086-7

**Published:** 2017-09-04

**Authors:** Robin A. Vora

**Affiliations:** Co-Chair of Ophthalmology, Kaiser Permanente, Northern California, Oakland, CA 94611 USA

**Keywords:** Vascular endothelial growth factor, Anti-VEGF, Choroidal Neovascularization, CNV, Bevacizumab, Ranibizumab, Aflibercept

## Abstract

This is a review of a book entitled, “Anti-VEGF Use in Ophthalmology,” written by Jay Duker, MD and Michelle Liang, MD. It is an excellent description of the history and development of anti-VEGF agents as well as an up-to-date and thorough account of their many clinical uses in Ophthalmology.

## Background

The development of anti-VEGF therapy has been one of the greatest achievements in Ophthalmology. These therapies have revolutionized the management of nearly every common retinal disease and have forever altered the prognosis for these patients, allowing most to retain useful vision throughout their life. The book “Anti-VEGF Use in Ophthalmology,” written by Jay Duker, MD and Michelle Liang, MD, is an outstanding review of the history, development and clinical use of anti-VEGF agents. It is a well-worth read for all residents, retina fellows, ophthalmic nurses, assistants and technicians.

## Main text

The book “Anti-VEGF Use in Ophthalmology” is a concise, up-to-date and through review of anti-VEGF therapy, from conception to clinical use. The book is organized in a very thoughtful fashion and flows effortlessly. Chapters are authored by some of the most influential retinal specialists in the field. Mindful readers will find tons of pearls embedded in their writings. The images are of the highest quality, allowing the reader to easily visualize and understand their teaching points.

Section one of the book details the fascinating chase to discover the “Factor X” responsible for angiogenesis and the seminal research responsible for the final identification of VEGF as the culprit. The ensuing chapters discuss currently available intravitreal anti-VEGF agents, from Pegaptanib to Aflibercept. The chapter on intravitreal delivery is in particular a must read, as it is a current evidence-based review on all aspects of intravitreal injections, now one of the most commonly done procedures in all of medicine. The many considerations regarding injection anesthesia and infection control are pondered in a thoughtful and academic fashion. Focus then turns to a broad discussion of potential new therapies and delivery systems.

The second section reviews all essential clinical uses of anti-VEGF therapy. Retinal disease is covered in earnest. All of the most ground breaking anti-VEGF clinical studies of the last decade are succinctly reviewed, encompassing macular degeneration, secondary choroidal neovascularization, diabetic retinopathy, retinal vein occlusion, and retinopathy of prematurity. Real life case images are of the highest quality and are generously included to help emphasize key teaching points. There are sections on non-retinal use of anti-VEGF agents as well. It was noteworthy to see how this therapy has also impacted the world of cornea, glaucoma, and uveitis.

## Conclusion

The book “Anti-VEGF Use in Ophthalmology” is a phenomenal read for medical students, residents and fellows interested in the field of Ophthalmology. Ancillary ophthalmic staff will also find this useful, helping them to understand the “why” as they go about their daily patient care duties. Finally, practicing ophthalmologists and retina specialists will find the book to be a concise yet thorough review, one that they can quickly read to relearn lost information and pick up new pearls of wisdom. I would strongly recommend this book to anyone interested in Ophthalmology.

## Abbreviation

VEGF: vascular endothelial growth factor

